# Isosteviol Derivative Inhibits Osteoclast Differentiation and Ameliorates Ovariectomy-Induced Osteoporosis

**DOI:** 10.1038/s41598-018-29257-1

**Published:** 2018-07-25

**Authors:** Huey-En Tzeng, Po-Hao Huang, Chun-Hao Tsai, Gregory J Tsay, Yi-Ju Lee, Tsurng-Juhn Huang, Tzu-Hung Lin, Ying-Ming Chiu, Yi-Ying Wu

**Affiliations:** 1 Taipei Cancer Center, Taipei Medical University, Taipei, Taiwan; 2 Graduate Institute of Cancer Biology and Drug Discovery, College of Medical Science and Technology, Taipei Medical University, Taipei, Taiwan; 3 Department of Internal Medicine, Division of Hematology/Oncology, Taipei Medical University – Shuang Ho Hospital, Taipei, Taiwan; 4 Department of Internal Medicine, School of Medicine, China Medical University Hospital and China Medical University, Taichung, Taiwan; 5 Division of Immunology and Rheumatology, Department of Internal Medicine, China Medical University Hospital, Taichung, Taiwan; 6 Department of Orthopedics, School of Medicine, China Medical University Hospital and China Medical University, Taichung, Taiwan; 7 Institute of Biochemistry, Microbiology and Immunology, Chung Shan Medical University, Taichung, Taiwan; 8 Department of Biochemistry, China Medical University, Taichung, Taiwan; 9 Material and Chemical Research Laboratories, Industrial Technology Research Institute, Chutung, Hsinchu County, Taiwan; 10 Division of Allergy, Immunology & Rheumatology, Changhua Christian Hospital, Changhua, Taiwan; 11 Department of Nursing, College of Medicine & Nursing, Hungkuang University, Taichung, Taiwan; 12 Department of Medical Laboratory Science and Biotechnology, China Medical University, Taichung, Taiwan; 13 Chinese Medicine Research Center, China Medical University, Taichung, Taiwan; 14 Research Center for Chinese Herbal Medicine, China Medical University, Taichung, Taiwan

## Abstract

NC-8 (ent-16-oxobeyeran-19-N-methylureido) is an isosteviol-derived analogue with multiple biological effects, including anti-inflammation and anti-bacterial activities and inhibition of HBV viral surface antigen gene expression. In this study, we explored the effects of NC-8 on the formation of osteoclasts from RAW 264.7 cells. We found that NC-8 exerts the novel effect of inhibiting osteoclast-like cell formation. Our experiments showed that RANKL-induced ERK, p38, and JNK phosphorylation were inhibited by NC-8. An ovariectomy-induced osteoporosis animal model was used to examine the protective effects of oral treatment with NC-8. Serum analysis was used to examine markers of osteoblasts, osteoclasts, and renal and hepatic function in rats. Micro CT scanning and histological analysis were used to measure bone loss in ovariectomized rats. Oral administration of NC-8 effectively decreased excess bone resorption and significantly antagonized trabecular bone loss in ovariectomized rats. Serum analysis of C-terminal telopeptide of type-I collagen, an osteoclast marker, also showed that NC-8 administration inhibited excess bone resorption. Furthermore, serum analysis showed that renal and liver function were not affected by these doses of NC-8 during long-term treatment. Our results demonstrate that NC-8 inhibits osteoclast differentiation and effectively ameliorates ovariectomy-induced osteoporosis.

## Introduction

Bone homeostasis is elaborately maintained by a balance involving bone formation and bone resorption. Osteoclasts, which are confirmed to be monocyte-macrophage–derived multinucleated cells, are uniquely specialized to carry out bone resorption^1^. As a member of the tumor necrosis factor (TNF) superfamily, RANKL binds to RANK on osteoclast precursors and stimulates their differentiation and fusion into mature osteoclasts via the activation of ERK, p38, and NF-κB^2,3^. Abnormal RANKL-induced osteoclast activation induces bone-related disease, such as post-menopausal osteoporosis^4–6^, rheumatoid arthritis^6–8^, Paget’s disease^6,8^, and osteolytic bone metastases^9–11^.

Isosteviol (ent-16-oxobeyeran-19-oic acid), an ent-beyerane tetracyclic diterpenoid, is a stevioside compound that is obtained via acid hydrolysis. Isosteviol derivatives exert a variety of biological effects, including anti-hypertension, anti-inflammation, anti-diarrhea, anti-cancer, anti-viral, and anti-bacterial activity^12–16^. NC-8, an isosteviol derivative, has been demonstrated to effectively inhibit the secretion of hepatitis B virus (HBV) surface antigens and HBV core antigens and inhibit the host TLR2/NF-κB signaling pathway^14^. However, the effect of NC-8 on RANKL activity through the ERK, p38, and NF-κB pathways has not been identified in the existing literature. In this study, we investigated how the isosteviol-derived novel compound NC-8 affects the differentiation of osteoclasts.

## Results

### Cytotoxicity of NC-8 in RAW 264.7 cells

**Figure 1:**
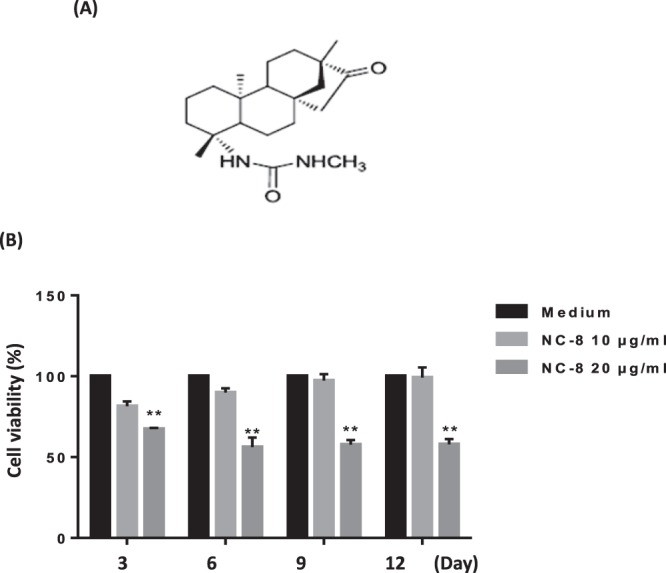
RAW 264.7 cells were treated with NC-8 to determine cytotoxicity. (**A**) NC-8 structural formula. The molecular weight of NC-8 is 346.51. (**B**) Cell viability tests were performed by MTT detection. RAW 264.7 cells were cultured at different concentrations of NC-8. After 3, 6, 9, and 12 days, MTT reagents were added to each well, and the absorbances were measured at 550 nm; the cell viability was then calculated. The results are expressed as the mean ± S.E.M. of four independent experiments. *p < 0.05, **p < 0.005 compared with the control group.

First, we investigated the toxicity of NC-8 (Fig. 1A) in RAW 264.7 cells and identified the appropriate concentration to use in the subsequent experiments. RAW 264.7 cells were treated with increasing concentrations (0, 1, 5, 10, 20, 40, and 80 μg/ml) of NC-8 for 72 h, and cell viability was analyzed using the MTT assay. The inhibitory concentration (IC_50_) was 40 μg/ml (Supplementary Fig. 1). We then tested the cytoxicity of NC-8 for longer periods of treatment. As shown in Fig. 1C, NC-8 treatment at the concentration of 20 μg/ml showed significant cytotoxicity after 6 days. Therefore, we chose a concentration of 10 μg/ml for use in our experiments.

### NC-8 inhibits RANKL-induced osteoclast differentiation

**Figure 2:**
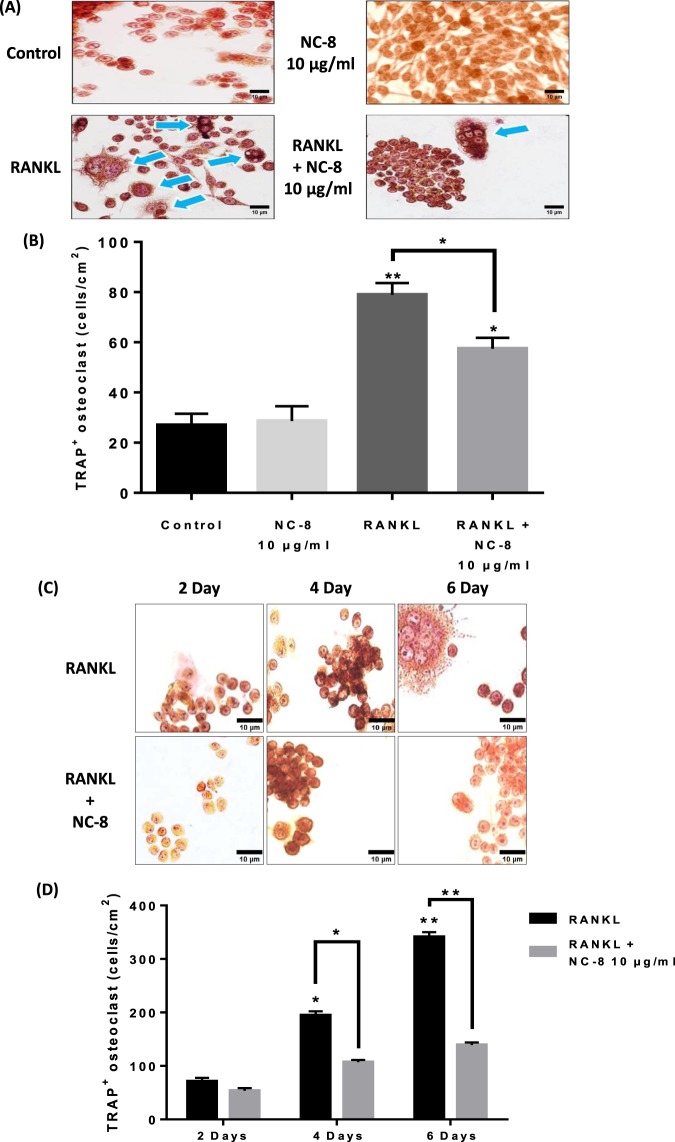
The inhibitory effects of NC-8 on RANKL-induced osteoclast differentiation. (**A**,**B**) RAW 264.7 cells were incubated in 24-well plates containing 1 × 10^4^ cells and treated with 100 ng/ml RANKL or 10 μg/ml, 20 μg/ml NC-8 for 6 days. After TRAP staining, the samples were examined under a 400X microscope. Arrowheads indicate the formed osteoclasts. Cell morphology was examined by light microscopy, and the number of TRAP-positive multinuclear cells was quantified. (**C**,**D**) RAW 264.7 cells were incubated in 24-well plates containing 1 × 10^4^ cells; the cells were then treated with 100 ng/ml RANKL or 10 μg/ml or 20 μg/ml NC-8 for 2, 4, or 6 days. After TRAP staining, the samples were examined under a 400 X microscope. Arrowheads indicate the formed osteoclasts. Cell morphology was examined by light microscopy, and the number of TRAP-positive multinuclear cells was quantified. The results are expressed as the mean ± S.E.M. of four independent experiments. *p < 0.05, **p < 0.005 compared with the control group.

The effects of NC-8 on RANKL-induced osteoclast differentiation were evaluated with TRAP staining. RAW 264.7 cells were cultured with various concentrations (10 or 20 μg/ml) of NC-8 in the presence of RANKL for 6 days. The results showed that incubation with 100 ng/ml RANKL and 10 μg/ml NC-8 reduced RANKL-induced osteoclast differentiation. Treatments comprising 100 ng/ml RANKL and 10 μg/ml NC-8 were cultured for 2, 4, and 6 days, and we found that 10 μg/ml NC-8 significantly inhibited RANKL-induced osteoclast differentiation at different times (Fig. 2A–D).

### NC-8 inhibits RANKL-induced osteoclast differentiation signaling pathways

We used Western blotting to investigate the main signaling pathways (NF-κB and MAPK) that are closely associated with osteoclast differentiation. Previous studies have shown that the three major subfamilies of MAPKs (p38, ERK1/2, and JNK) play pivotal roles in osteoclast development downstream of RANK signaling. Furthermore, NF-κB pathway activation is also crucial for osteoclast formation^17,18^.

To elucidate the mechanism by which NC-8 inhibits RANKL-induced osteoclast differentiation and function, we investigated whether the aforementioned signaling pathways were involved in the NC-8 mediated inhibition of RANKL-induced osteoclast differentiation.

**Figure 3:**
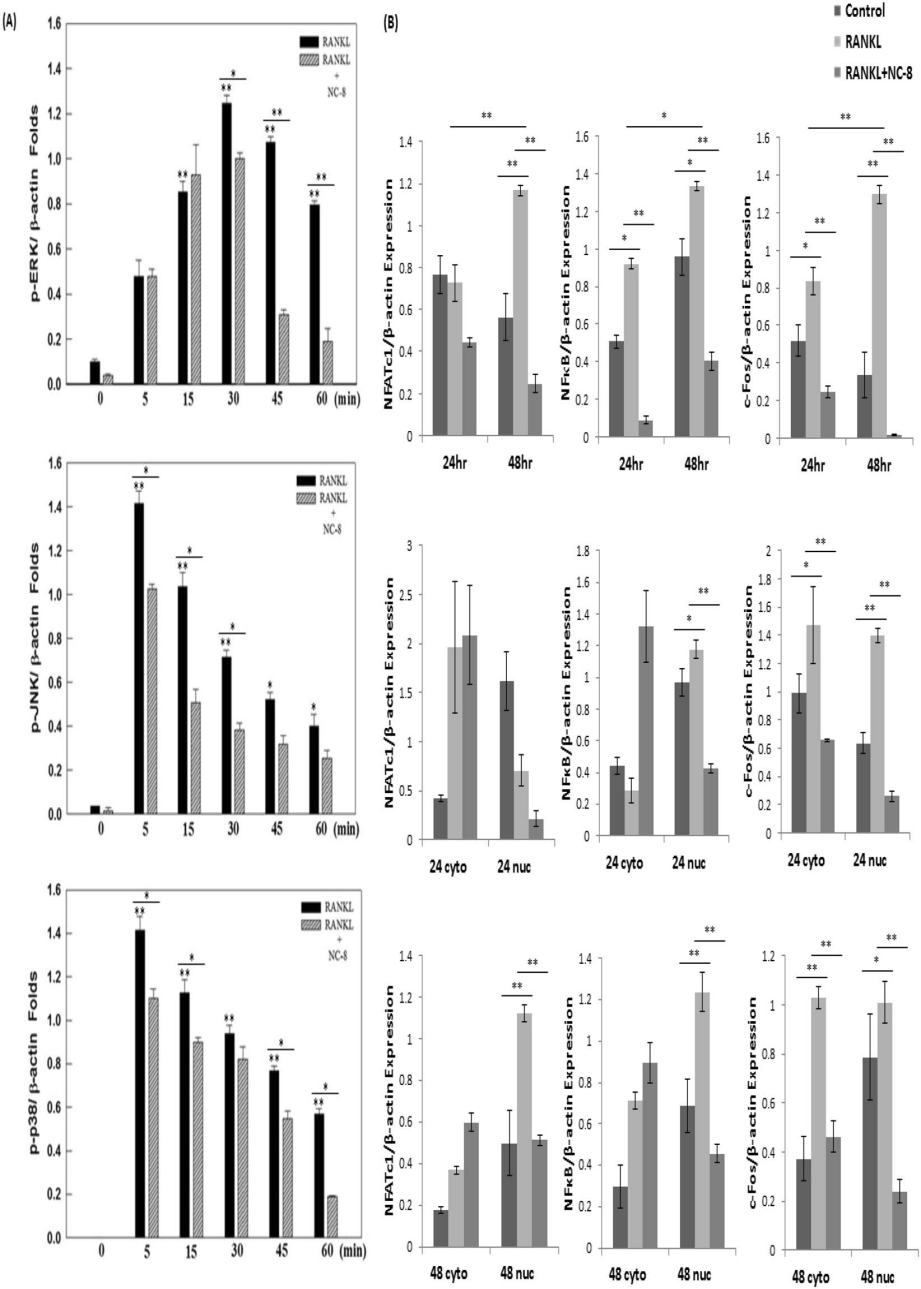
The inhibitory effects of NC-8 on the RANKL-induced osteoclast differentiation signaling pathway. (**A** and **B**) RAW 264.7 cells were treated with 100 ng/ml RANKL or with 10 μg/ml NC-8, and the protein was collected after the reaction. Western blot was used to detect MAPK pathway and downstream NFATc1, c-Fos and NF-κB protein molecules. (**A**) Relative protein expressions analysis of proteins related to the MAPK signaling pathway, including p-ERK, p-JNK, and p-p38. β-actin was used as a loading control. (**B**) Relative protein expressions analysis of proteins related to the NF-κB signaling pathway, including p65, NFATc1 and c-Fos. β-actin was used as a loading control. Relative expression levels of p65, NFATc1 and c-Fos protein in the cytoplasm and in the nuclear fractions. β-actin and HDAC1 were used as loading controls. The results are expressed as the mean ± S.E.M. of four independent experiments. *p < 0.05, **p < 0.005 compared with the control group.

RAW 264.7 cells were treated with 100 ng/ml RANKL and 10 μg/ml NC-8 for 5–60 min. As shown in Fig. 3A, NC-8 inhibited the phosphorylation of MAPKs (p38, ERK1/2, and JNK), and the original image of the immunoblotting in (Supplementary Fig. 5). We then examined the effect of NC-8 on the inhibition of c-Fos, NFATc1, and NF-κB p65 protein expression. As expected, Western blotting analysis confirmed that treatment with NC-8 for 24 or 48 h had significant effects on the downstream levels of c-Fos, NFATc1, and NF-κB p65 protein expression (Fig. 3B), and the original image of the immunoblotting in (Supplementary Fig. 5). Given the indispensable role of NF-κB in RANKL-induced osteoclastogenesis, we hypothesized that NC-8 exerted its inhibitory function through NF-κB pathway regulation. To validate this hypothesis, we evaluated the expression of p65, c-Fos, NFATc1 in response to NC-8 treatment. We first validated that RANKL activated the NF-κB pathway, as indicated by decreases in p65, c-Fos, and NFATc1 levels (Fig. 3B). In the nuclear fraction, p65 was upregulated by RANKL, and this was counteracted by NC-8 treatment (Fig. 3B). These data supported our assumption that the regulatory role of NC-8 was linked to its inhibition of the NF-κB pathway.

Taken together, these results suggest that NC-8 inhibits osteoclast differentiation by inhibiting MAPK signaling and affecting the c-Fos, NFATc1, and NF-κB pathways.

### NC-8 inhibits osteoclast bone resorption activity

**Figure 4:**
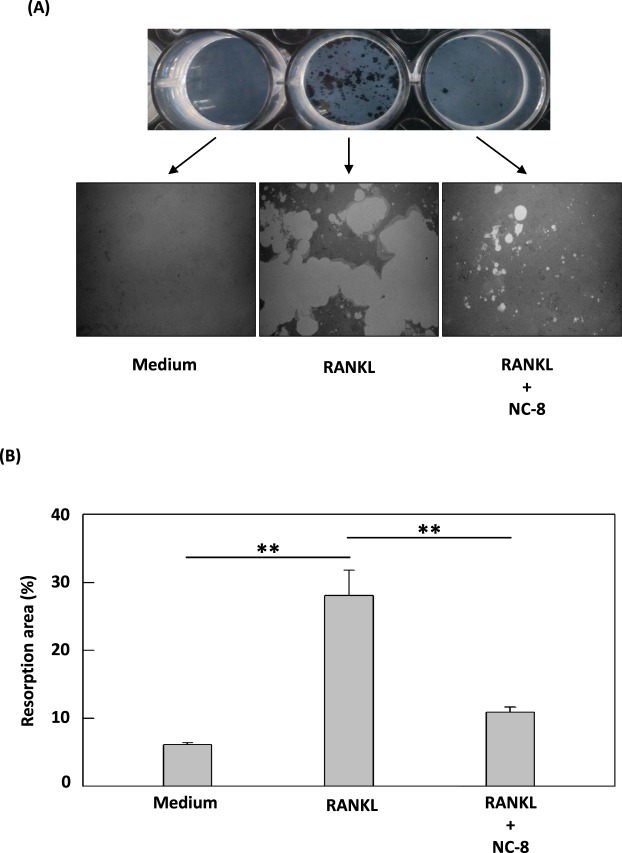
The inhibitory effects of NC-8 on osteoclast bone resorption activity. (**A** and **B**) RAW 264.7 cells were treated with 100 ng/ml RANKL or 10 μg/ml NC-8 and cultured on a Corning Osteo Assay Surface culture plate. After RANKL treatment for 4 days and subsequent NC-8 treatment for 8 days, the area of resorption was observed under a 40 X microscope. The results are expressed as the mean ± S.E.M. of four independent experiments. *p < 0.05, **p < 0.005 compared with the control group.

To understand whether NC-8 reduces RANKL-induced osteoclast bone erosion, we used commercial calcium phosphate apatite as a resorption substrate to determine the degree of pit formation caused by RAW 264.7 cells compared to cell cultures without differentiation agents. Lacunar resorption was observed in RAW 264.7 cells treated with NC-8 for 12 days on calcium phosphate apatite plates. Our results clearly demonstrate that NC-8 not only reduces the formation of osteoclasts but also inhibits their dissolution (Fig. 4A,B).

### Oral administration of NC-8 inhibited OVX-induced bone loss in rats

We further examined the effects of NC-8 on OVX-induced osteoporosis in rats. NC-8 was administered via gastric intubation every 3 days at concentrations of 1, 3, and 10 mg/kg (Table 1). No significant changes in body weight (when compared with rats treated with vehicle) were observed in OVX rats after the administration of NC-8 (Supplementary Fig. 2).

**Table 1:** Research and design of osteoporosis animals.

Group	Test article	Route	No. of Rats	Dose level	Dosing frequency
Sham	Vehicle	p.o.	12	1 ml/kg	Once per 3 days
Vehicle control	Vehicle	p.o.	13	1 ml/kg	Once per 3 days
Treatment- NC-8-1 mg/kg	NC-8	p.o.	12	1 mg/kg	Once per 3 days
Treatment- NC-8-3 mg/kg	NC-8	p.o.	12	3 mg/kg	Once per 3 days
Treatment- NC-8-10 mg/kg	NC-8	p.o.	12	10 mg/kg	Once per 3 days

**Figure 5:**
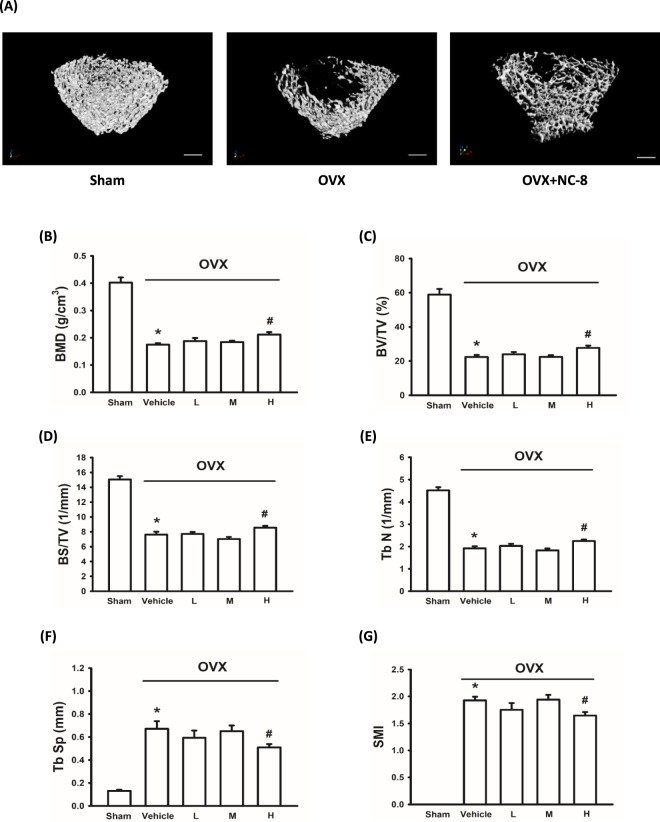
Oral administration of NC-8 inhibits ovariectomy-induced trabecular bone loss in isolated femur. Distilled water or NC-8 (1, 3 and 10 mg/kg each day for 3 days) was orally administered to OVX rats via gastric intubation for 84 days (once each 3 days). The rats were killed on Day 91. The femur and tibia were collected for micro-CT analysis. (**A**) Representative (transaxial view) images showed that the administration of NC-8 (10 mg/kg each 3 days) inhibited the trabecular bone loss in the femur of OVX-rats (scale bar = 1 mm). The quantitative data are shown in (**B**–**G**). Treatment with NC-8 (10 mg/kg each 3 days) also inhibited the OVX-induced decreases of trabecular BMD (**B**), bone volume (**C**), bone surface (**D**), bone number (**E**), as well as the increase of trabecular bone separation (F) and trabecular thickness (**G**). Values represent the mean ± S.E.M. (n = 5–9). *p < 0.05, compared with the sham-operated group (Sham). ^#^p < 0.05 compared with the OVX-control group (Con). (L: 1 mg/kg each 3 days) (M: 3 mg/kg each 3 days) (H: 10 mg/kg each 3 days).

The rats were killed on Day 91, and serum and femurs were collected for further analysis. The trabecular bone of the secondary spongiosa area of the proximal femur (Fig. 5) was scanned using μCT. μCT analysis showed that the administration of 10 mg/kg NC-8 every 3 days exerted significant protective effects on the trabecular bone of OVX rats as evidenced by bone mineral density and several bone-morphometric indices (e.g., bone volume, bone surface, and trabecular number and separation).

**Figure 6:**
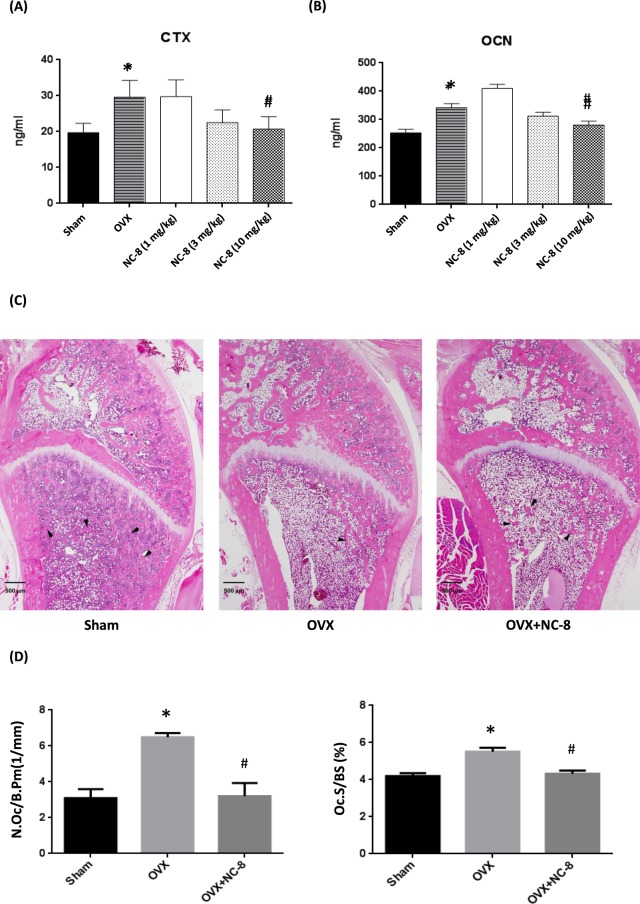
Effects of NC-8 on the OVX-induced up-regulation of serum markers for bone resorption. (**A**) NC-8 (1, 3 and 10 mg/kg each 3 days) significantly antagonized the OVX-induced increase of serum levels of osteoclastic markers. (**B**) NC-8 (1, 3 and 10 mg/kg each 3 days) also antagonized the compensatory increase of serum osteocalcin. Values represent the mean ± S.E.M. *p < 0.05 compared with the sham group (Sham). ^#^p < 0.05 compared with the OVX group (OVX). (**C**) H&E staining shows that NC-8 (10 mg/kg each 3 days) antagonized OVX-induced trabecular bone loss. Black arrows indicate the cancellous bone in secondary spongiosa. (**D**) Bone-resorption parameters N.Oc/B.Pm and Oc.S/BS were quantified using OsteoMeasure image-analysis software. (NC-8: H: 10 mg/kg each 3 days) *p < 0.05, compared with the sham group (Sham). ^#^p < 0.05 compared with the OVX group (OVX).

We then examined bone metabolic markers in serum. The serum analysis showed that OVX increased levels of the bone resorption marker CTX-I, and this was accompanied by a compensatory increase in the osteoblastic marker OCN, which is considered a feedback control mechanism when excess bone resorption occurs^19^. NC-8 administration significantly antagonized OVX-induced up-regulation of bone CTX-1 and OCN (Fig. 6A,B). OVX also increased levels of the osteoclast marker TRAP-5b and the osteoblast marker ALP. NC-8 (high concentration) treatment significantly reduced the serum level of TRAP-5b, and this was followed by a reduction in the serum level of ALP (Supplementary Fig. 3).

The histological analysis also showed that treatment with NC-8 inhibited OVX-induced trabecular bone loss (Fig. 6C). N.Oc/B.Pm and Oc.S/BS were significantly lower in the NC-8 (H concentration) group than in the OVX group (Fig. 6D). These results indicate that the oral administration of NC-8 can ameliorate OVX-induced osteoporosis by inhibiting excess osteoclast-mediated bone resorption. Furthermore, NC-8 treatment for 84 days did not affect serum GOT and GPT in the liver or BUN and Cre in the kidney (Supplementary Fig. 4).

## Discussion

Morphogenesis and bone remodeling involves the synthesis of bone matrix by osteoblasts and the coordinated resorption of bone by osteoclasts. Osteoclasts are multinucleated giant cells that differentiate from precursors of the monocyte/macrophage cell lineage. When the activity of osteoclasts is greater than that of osteoblasts, the rate of bone resorption becomes greater than that of synthesis, resulting in osteoporosis.

There are active compounds derived from natural products have been reported to possess inhibitory effects on osteoclast differentiation and function for new drug candidates^20^. NF-κB is an important signal mediator for inflammatory and immune reactions and is a major transcription factor for RANKL activated osteoclastogenesis^21^. In addition to NF-κB pathway, three members of mitogen-activated protein kinases (MAPK)s,ERK, JNK, and p38 are also activated by RANKL stimulation and play important roles in osteoclast differentiation^22^. NFATc1 is a master regulator in osteoclastogenesis, and its induction is a critical step that determines the cell fate of osteoclasts^23,24^. NFATc1 is a key target gene of NF-κB at the early phase of osteoclastogenesis^25^. NF-κB subunits p50 and p65 are recruited to the NFATc1 promoter after RANKL stimulation and also showed that the overexpression of NF-κB activated NFATc1 promoter activity in a luciferase reporter assay^25^.

The natural product derived compound proved inhibiting osteoclasogenesis by modulate RANKL-NF-κB and/or (MAPKs ERK, p38 and JNK) pathways, include flavonoids, glucosides, alkaloids, coumarins, lignans, terpenoids, polyphenols and ect^20^ However, the anti-bone resorption effect by isosteviol derivatives has not reported in the literature.

Previous studies have revealed the biological effects of isosteviol derivatives, including anti-hypertension, anti-inflammation, anti-diarrhea, anti-cancer, anti-viral, and anti-bacterial activity^12–16,26^. Specifically, isosteviol derivatives were found to inhibit the NF-κB signaling pathway^14,26,27^, which is closely associated with osteoclast differentiation^18,28,29^. Therefore, we set out to investigate the effects of an isosteviol derivative, NC-8, in RANKL-induced osteoclastogenesis, which is activated through the NF-κB and MAPK signaling pathways. Here, we have demonstrated the anti-osteoporosis effects of an isosteviol derivative for the first time. With adequate concertation of 10 µg/ml, we also first proved anti-osteoporosis effect of isosteviol derivative in ovariectomy-induced osteoporosis animal model in the literature.

In this study, we first determined the appropriate treatment concentration of NC-8 by MTT assay (Fig. 1). As evaluated by TRAP staining, 10 µg/ml of NC-8 was sufficient for the inhibition of RANKL-induced osteoclast differentiation (Fig. 2). By Western blotting, we showed that NC-8 inhibited osteoclast differentiation by inhibiting the MAP kinase pathway and the downstream proteins c-Fos and NFATc1 (Fig. 3), suggesting that NC-8 acts via a similar mechanism to paeonol^30^. In addition to inhibiting RANKL-induced osteoclast differentiation, NC-8 also inhibited the bone resorption activity of mature osteoclasts (Fig. 4).

Based on μCT analysis, NC-8 administration (10 mg/kg, every 3 days) exerted significant protective effects on the trabecular bone of OVX rats. Compared to control, there was a 20% increase in BMD, a 23% increase in BV/TV, and a 15.85% increase in BS/TV (Table 2). The protective effects of isosteviol derivatives on OVX-induced osteoporosis act mainly by inhibiting the loss, rather than increasing the thickness, of spongy bone. NC-8 inhibited decreases in BMD, BV/TV, BS/TV, and Tb N, and slowed the increases in Tb Sp (Fig. 5). NC-8 exerted a smaller effect on OVX-induced decreases in trabecular thickness, which indicated that the effects of NC-8 were mainly due to the inhibition of excessive osteoclast resorption (Fig. 5).

**Table 2:** Summarized micro-CT results for the effects of NC-8 administration on ovariectomy-induced bone loss.

	Sham	OVX-Vehicle	L	M	H
BMD	0.38 ± 0.03	0.17 ± 0.01^a^	0.19 ± 0.01	0.18 ± 0.01	0.21 ± 0.01^b^
BV/TV	58.91 ± 2.55	22.31 ± 1.32^a^	23.90 ± 1.42	22.47 ± 0.93	28.64 ± 1.56^b^
BS/TV	15.04 ± 0.36	7.13 ± 0.34^a^	7.71 ± 0.27	7.02 ± 0.28	8.49 ± 0.24^b^
Tb.N	4.52 ± 0.11	1.88 ± 0.10^a^	2.03 ± 0.10	1.83 ± 0.08	2.25 ± 0.06^b^
Tb.Sp	0.14 ± 0.01	0.66 ± 0.06^a^	0.59 ± 0.06	0.65 ± 0.05	0.50 ± 0.03^b^
Tb.Th	0.130 ± 0.004	0.115 ± 0.002^a^	0.118 ± 0.001	0.123 ± 0.002	0.124 ± 0.003

BMD: bone mineral density (g/cm^3^); BV/TV: bone volume ratio (%); BS/TV: bone surface ratio (1/mm); Tb.N: trabecular bone number (1/mm); Tb.Sp: trabecular bone separation (mm); Tb.Th: trabecular bone thickness (mm); the values represents the means ± S.E.M. ^a^P < 0.05 compared with the sham group. ^b^P < 0.05 compared with the OVX-vehicle group.

Oral treatment with NC-8 significantly antagonized estrogen deficiency–induced osteoporosis in rats. We converted the drug dosage used in rats to the equivalent human dosage for use in future applications^31^ as follows: 1, 3 and 10 mg/kg doses used in rats are equivalent to 10, 30 and 100 mg/60 kg respectively, in adult humans. The rat dosage divided by 6.2 is equivalent to the human dosage; i.e., 10 mg/kg of NC-8 in rats is equivalent to 1.61 mg/kg in humans. For a 60-kg human, the dosage used would be 1.61 × 60 = 96.77 mg (approximately 100 mg), which is a reasonable dose that would be suitable for use in clinical trials in the future.

The development of new osteoclast-targeting small molecule compounds is an important step in accelerating osteoclast research for the prevention or treatment of bone diseases and for bone regenerative medicine. Our present study reveals oral treatment with NC-8 every three days is sufficient to antagonize estrogen deficiency–induced osteoporosis in rats. Thus, NC-8 may be beneficial for the prevention of menopause-induced osteoporosis.

## Methods

### Cell lines and materials

We used the RAW 264.7 murine monocytic/macrophagic cell line as model system of osteoclastogenesis. These cells differentiate into osteoclast-like cells in the presence of RANKL. RAW 264.7 cells derived from a tumor induced by the Abelson murine leukemia virus were purchased from the American Type Culture Collection (ATCC; Rockville, MD). For osteoclast culture, the RAW 264.7 cells were seeded in a 24-well plate at a density of 1 × 10^4^ cells/well and were cultured for 7 days in the presence of 100 ng/ml RANKL as a positive control as our previously described^32^. RANKL was purchased from PeproTech (Rocky Hill, CT, United States). Rabbit antibody against NFATc1 (D15F1) was purchased from Cell Signaling (Danvers, MA, United States). The human osteoblast-like cell line MG-63 (CRL-1427) was purchased from the American Type Culture Collection. Cells were cultured in α-MEM supplemented with 10% FBS and antibiotics (100 U/ml penicillin and 100 μg/ml streptomycin)^30^.

### Cell viability

Cell viability was determined using the 3-[4,5-dimethylthiazol-2-yl]-2,5 diphenyltetrazoliumbromide (MTT) assay. After treatment with NC-8 for 3 days, MTT (0.5 mg/ml) was added to each well, and the cells were incubated for 2 h at 37 °C. After shaking at room temperature for 5 min, the absorbance of each well was determined at 570 nm using a microplate reader (Bio-Tek, Winooski, VT) as our previously described^32^.

### TRAP staining

Osteoclast formation was measured by quantifying cells that were positively stained with TRAP (Acid Phosphatase Kit 387-A; Sigma-Aldrich, St. Louis, MO). Briefly, specimens were fixed for 15 mins and then stained with naphthol AS-BI phosphate and a tartrate solution for 1 h at 37 °C, followed by counterstaining with a hematoxylin solution. Control cells are positively stained red; therefore, TRAP-positive multinuclear cells with three or more nuclei were regarded as osteoclasts and were counted under an inverted-phase contrast microscope. We also used a higher concentration of 1 M tartrate (for a final concentration of 20 mM), instead of the tartrate solution provided in the kit to suppress background phosphatase activity. By increasing the tartrate concentration, the staining of the control cells is suppressed, and the RANKL-treated cells remain positively stained. The total number of TRAP-positive cells and the number of nuclei per TRAP-positive cell in each well were counted. The morphological features of osteoclasts were also photographed as our previously described^32^.

### Bone resorption assay

To confirm the bone resorption ability of differentiated osteoclasts, we cultured RAW 264.7 macrophages in 24-well plates coated with artificial bone substrate (Bone Cell Culture System; Corning) under the same culture conditions as described above. After RANKL treatment for 4 days and subsequent NC-8 treatment for 8 days, the cells were washed with PBS and were then detached in the presence of 5% sodium hypochlorite for 5 min. The area of resorption was measured using microscopy (Olympus), and the pits were photographed as our previously described^32^.

### Preparation of cytoplasmic and nuclear extracts

Cells were washed twice with 1 × PBS and collected using a rubber policeman. Cell pellets were lysed in 200 µl of buffer I [10 mM HEPES (pH 7.9), 1.5 mM MgCl_2_, 10 mM KCl, 0.2 mM PMSF, 0.5% NP-40, 10 ug/ml leupeptin, 0.5 mM DTT and protease inhibitors] and incubated for 5 min. Nuclear extracts were separated by centrifugation at 14000 rpm for 15 mins; the supernatant contained the cytoplasmic extracts. The nuclear fraction was washed with buffer I without NP-40 three times and was then centrifuged at 14000 rpm for 5 min. Pellets (nuclear extracts) were then resuspended in 10~20 µl of buffer II [20 mM HEPES (pH 7.9), 1.5 mM MgCl_2_, 420 mM NaCl, 25% (v/v) glycerol, 0.2 mM EDTA, 0.2 mM PMSF and 0.5 mM DTT] and then placed on ice for 15 min. The samples were then centrifuged at 14000 rpm for 15 min at 4 °C, and the supernatant was used as the nuclear extract^33^.

### Western blot analysis

Proteins were resolved on SDS-PAGE gels and were then transferred to Immobilon polyvinyldifluoride (PVDF) membranes. The blots were blocked with 5% non-fat dry milk in Tris-buffered saline containing 0.5% Tween-20 (TBST) for 1 h at room temperature and were then probed with rabbit anti-mouse antibodies against NF-κB, p-p38, p-ERK, p-JNK, p38, ERK, JNK, c-Fos, and NFATc1 (1:1000; all from Cell Signaling) for 1 h at room temperature. After three washes, the blots were incubated with a donkey anti-rabbit peroxidase-conjugated secondary antibody (1:1000) for 1 h at room temperature. The blots were visualized with enhanced chemiluminescence using Kodak X-OMAT LS film (Eastman Kodak). Quantitative data were obtained using a computing densitometer and Image J software as our previously described method^34,35^.

### Animals

All protocols complied with institutional guidelines and were approved by the Animal Care Committee of China Medical University. The rats were housed in the China Medical University Laboratory Animal Center under a 12-h light cycle at 21–23 °C with 60–85% humidity. The experimental rats were purchased from BioLASCO Taiwan Co. Ltd. (I-Lan, Taiwan). Seven-week-old female Sprague-Dawley rats (176–200 g) were used for the ovariectomy (OVX)-induced osteoporosis model.

### Ovariectomy (OVX) surgery and micro-computed tomography (μCT) analysis

The animal study was performed in female Sprague-Dawley rats^36^. Rats were ovariectomized bilaterally under Zoletil (Virbac, Carros, France)/Rompun (Bayer Animal Health GmbH, Leverkusen, Germany) anesthesia, and control rats were sham-operated for comparison. Rats were administered with antibiotics (Baytril, Bayer Animal Health GmbH) after surgery and left to recover for 7 days. All animals were housed under controlled conditions at room temperature (22 ± 1 °C) under a 12-h light-dark cycle. Distilled water or NC-8 was administered to the rats by gastric intubation (once every 3 days for 84 days). A high resolution X-ray microtomography apparatus (Skyscan 1176, Skyscan-Bruker, Kontich, Belgium) was used to analyze the bone mineral density and morphometric indices of trabecular bone^36^. On Day 91, the rats were killed, and their femurs were removed, fixed with 4% paraformaldehyde, and analyzed with μCT. Scanning was performed at 65 kVp and 387 μA with a 1-mm aluminum filter. Images of isolated femur were collected at a resolution of 9 μm/pixel. The sections were reconstructed using GPU-based scanner software (NRecon). Bone mineral density and trabecular morphometric indices were quantified in a defined cancellous bone area located 2–4 mm (225 sections) below the growth plate of the proximal end of the femur. The analysis was performed using CTAn analysis software. Trabecular morphology was evaluated by measuring the bone volume fraction (bone volume/tissue volume, BV/TV), ratio of bone surface to tissue volume (BS/TV), trabecular number (Tb N), trabecular thickness (Tb Th), and trabecular bone mineral density (BMD). Three-dimensional (3D) images were obtained using CTvox software^37^.

### Analysis of bone resorption, and serum osteoblastic, renal, and liver toxicity markers

Rats were anesthetized and killed at the end of the experiment, and a blood sample was quickly obtained from the left ventricle. Serum samples were prepared by centrifugation. Levels of C-terminal telopeptides of type-I collagen (CTX) were measured using the Serum Rat-Laps ELISA assay for the evaluation of bone resorption (Immunodiagnostic Systems, Boldon Colliery, Tyne & Wear, United Kingdom). Osteocalcin (OCN) levels were measured with a Serum Rat-Mid ELISA kit to evaluate osteoblastic activity (Immunodiagnostic Systems). Serum levels of TRAP-5b were measured by enzyme-linked immunosorbent assay (ELISA) (Immunodiagnostic Systems, AZ). Serum levels of ALP was measured according to the manufacturers’ instructions using a LabAssay ALP (p-nitrophenyl phosphate (pNPP) assay). Serum levels of glutamate oxaloacetate transaminase (GOT), glutamate pyruvate transaminase (GPT), blood urea nitrogen (BUN), and creatinine (Cre) levels were determined using an auto-dry chemistry analyzer (SPOTCJEM SP-4410, Arkray, Inc., Kyoto, Japan).

### Hematoxylin and eosin (H&E) staining of femur sections

Rat femurs were fixed in 4% paraformaldehyde at 4 °C for 48 h, decalcified in 10% Na2EDTA at 4 °C for 28 days, dehydrated in increasing concentrations of ethanol, and then embedded in paraffin. Serial longitudinal histological sections were cut at 6 μm and stained with a H&E staining kit. Images of the growth plate and proximal femur were photographed using a CX31 microscope (Olympus, Tokyo, Japan).

### Bone histomorphometry

Both static and dynamic bone-formation and bone-resorption parameters were evaluated by histomorphometric analysis. After μCT scanning, both the L4 vertebrae and tibias were embedded in methyl methacrylate (MMA), and 4- and 8-mm sections were obtained. Four-millimeter sections were prepared for TRACP staining to evaluate osteoclast number (N.Oc/B.Pm) and osteoclast surface (Oc.S/BS) Histomorphometric analysis was performed using OsteoMeasure image-analysis software (OsteoMetrics, Atlanta, GA, USA). Bone histomorphometric variables were expressed according to the report of the American Society of Bone and Mineral Research Nomenclature Committee^38^.

### Statistical analysis

All data were analyzed using SPSS 15.0 for Windows^39,40^. Each experiment was performed in duplicate, and average values were used for quantification. Data are expressed as the mean ± the standard error (SE) of at least three experiments. For statistical analyses of data containing more than three groups, we used one-way analysis of variance (ANOVA) followed by Tukey’s post hoc test. A p value of <0.05 was considered significant.

## Electronic supplementary material

Supplementary Information
