# Supplementary material for: Isosteviol Derivative Inhibits Osteoclast Differentiation and Ameliorates Ovariectomy-Induced Osteoporosis
Source: Sci Rep. 2018 Jul 25;8:11190. doi: 10.1038/s41598-018-29257-1 (PMC6060097; doi:10.1038/s41598-018-29257-1)
Supplement: Supplementary file 1 — Supplementary Information [file 41598_2018_29257_MOESM1_ESM.docx]

**Supplementary Information**

**Isosteviol Derivative Inhibits Osteoclast Differentiation and Ameliorates Ovariectomy-Induced Osteoporosis**

Huey-En Tzeng^1,2,3*^, Po-Hao Huang^4,5^, Chun-Hao Tsai^6*^, Gregory J Tsay^4,5^, Yi-Ju Lee^7^, Tsurng-Juhn Huang^8^, Tzu-Hung Lin^9^, Ying-Ming Chiu^10,11^, Yi-Ying Wu^12,13,14^

^1^  Taipei Cancer Center, Taipei Medical University, Taipei, Taiwan

^2^ Graduate Institute of Cancer Biology and Drug Discovery, College of Medical Science and Technology, Taipei Medical University, Taipei, Taiwan

^3^ Department of Internal Medicine, Division of Hematology/Oncology, Taipei Medical University – Shuang Ho Hospital, Taipei, Taiwan

^4^ Department of Internal Medicine, School of Medicine, China Medical University Hospital and China Medical University, Taichung, Taiwan

^5^ Division of Immunology and Rheumatology, Department of Internal Medicine, China Medical University Hospital, Taichung, Taiwan

^6^ Department of Orthopedics, School of Medicine, China Medical University Hospital and China Medical University, Taichung, Taiwan

^7^ Institute of Biochemistry, Microbiology and Immunology, Chung Shan Medical University, Taichung, Taiwan

^8^ Department of Biochemistry, China Medical University, Taichung, Taiwan

^9^ Material and Chemical Research Laboratories, Industrial Technology Research Institute, Chutung, Hsinchu County, Taiwan

^10^ Division of Allergy, Immunology & Rheumatology, Changhua Christian Hospital, Changhua, Taiwan

^11^ Department of Nursing, College of Medicine & Nursing, Hungkuang University, Taichung, Taiwan

^12^ Department of Medical Laboratory Science and Biotechnology, China Medical University, Taichung, Taiwan

^13^ Chinese Medicine Research Center, China Medical University, Taichung, Taiwan.

^14^ Research Center for Chinese Herbal Medicine, China Medical University, Taichung, Taiwan.

* Contributed equally

Address correspondence to:

Yi-Ying Wu, Ph.D.; E-mail: yyw@mail.cmu.edu.tw

Department of Medical Laboratory Science and Biotechnology, China Medical University, No. 91, Hsueh-Shih Rd., Taichung 404, Taiwan.

Chinese Medicine Research Center, China Medical University, Taichung 404, Taiwan.

Research Center for Chinese Herbal Medicine, China Medical University, Taichung 404, Taiwan.

Tel: +886 4 22053366 x7228 Fax: +886 4 22057414.


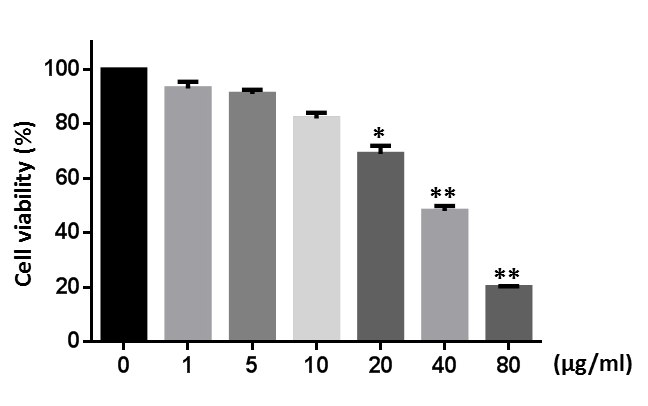


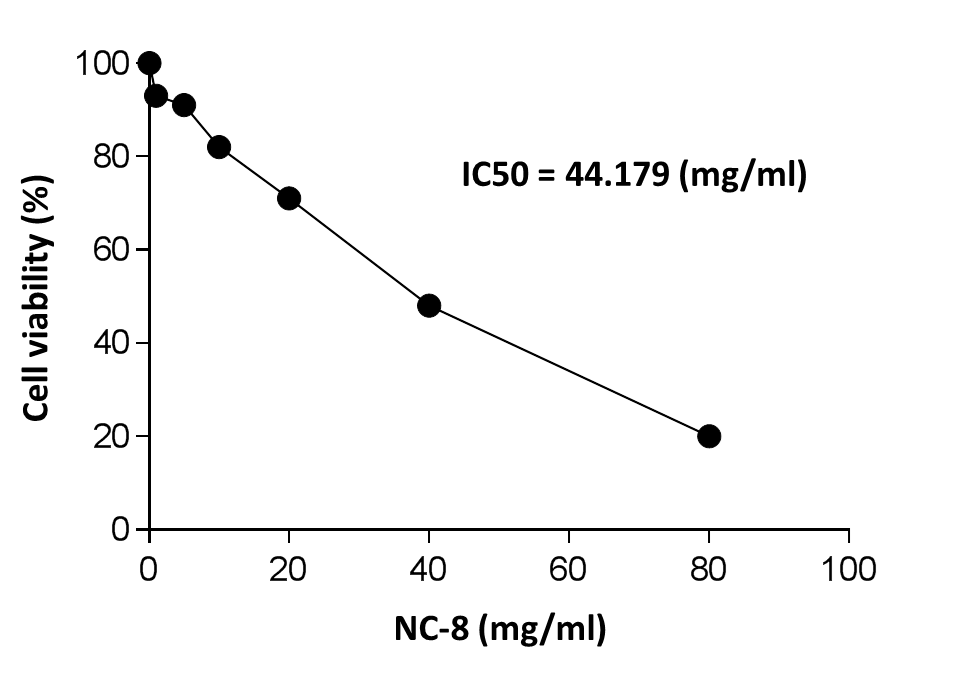


**Supplementary Figure 1. The inhibitory concentration (IC_50_) of NC-8**

RAW 264.7 cells were treated with increasing concentrations (0, 1, 5, 10, 20, 40, and 80 μg/ml) of NC-8 for 72 h, and cell viability was analyzed using the MTT assay. The results are expressed as the mean ± S.E.M. of four independent experiments. *p< 0.05, **p< 0.005 compared with the control group.


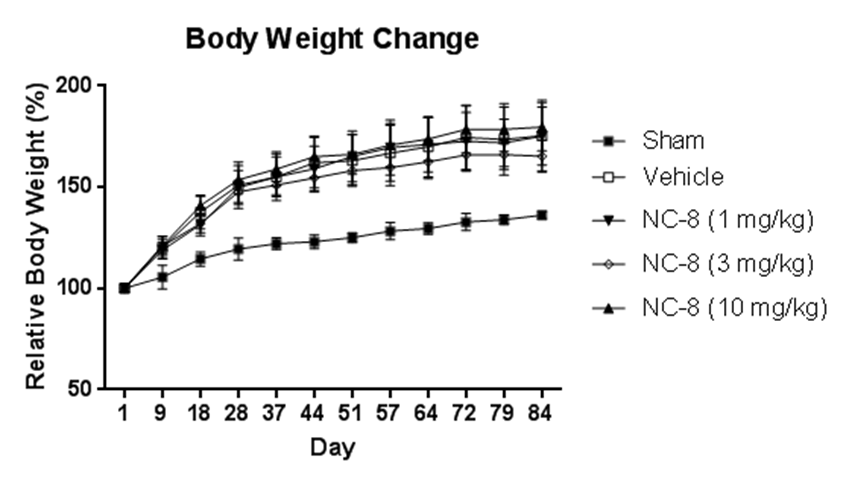


**Supplementary Figure 2. NC-8 exerts no significant effect on body weight of OVX-rats**

NC-8 (1, 3 and 10 mg/kg/ 3 days) or distilled water was orally administered to OVX rats via gastric intubation for 84 days (once/ 3 days). The body weight was recorded twice a week. The body weight of OVX rats increased compared with sham-operated rats.


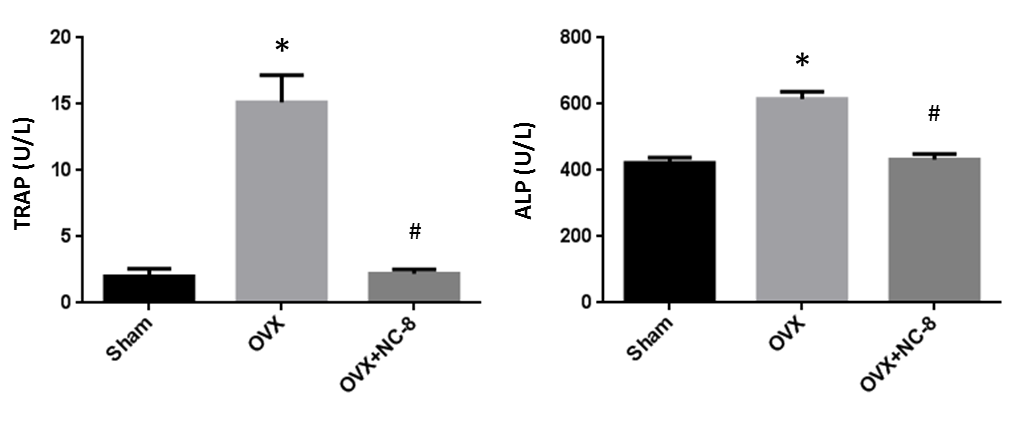


**Supplemental Figure 3. Effects of NC-8 on serum TRAP-5b and ALP levels**

Serum TRAP-5b and ALP levels were measured by ELISA or pNPP assay. Each value represents mean ± S.E.M. *, p<0.05 as compared with the sham group (Sham). #, p < 0.05 as compared with OVX group (OVX). (NC-8: H: 10 mg/kg/ 3 days)


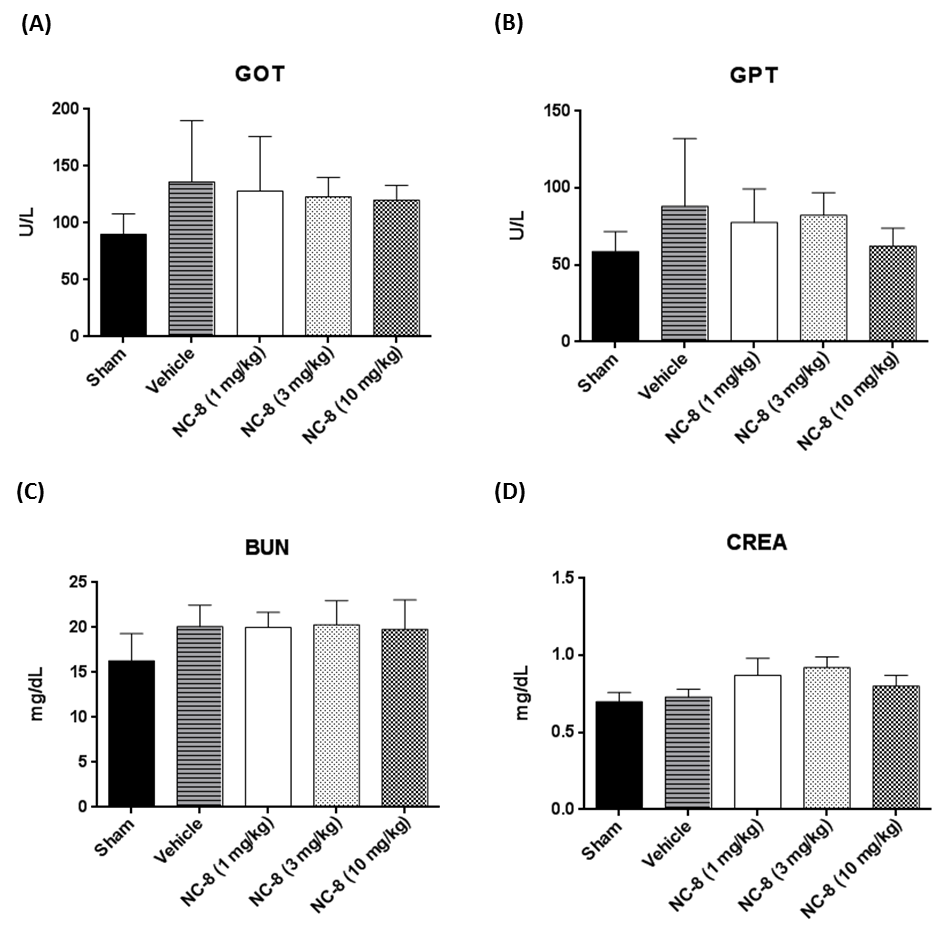


**Supplemental Figure 4. NC-8 exerts no toxic effect on renal or liver function**

Distilled water or NC-8 (1, 3 and 10 mg/kg/ 3 days) was orally administered to OVX rats via gastric intubation for 84 days (once/ 3 days). The serum of rats was collected and analyzed on Day-91. It was found that oral administration of NC-8 did not affect the levels of GOT (A) and GPT (B), which are the markers of liver function, and blood urea nitrogen (C), creatinine (D), which are markers of renal function. Each value represents mean ± S.E.M.


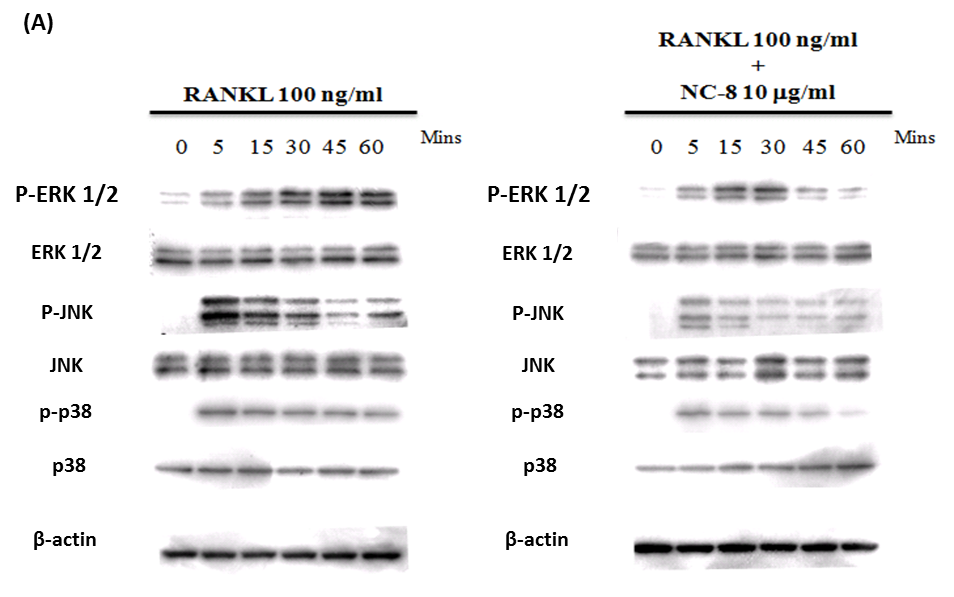


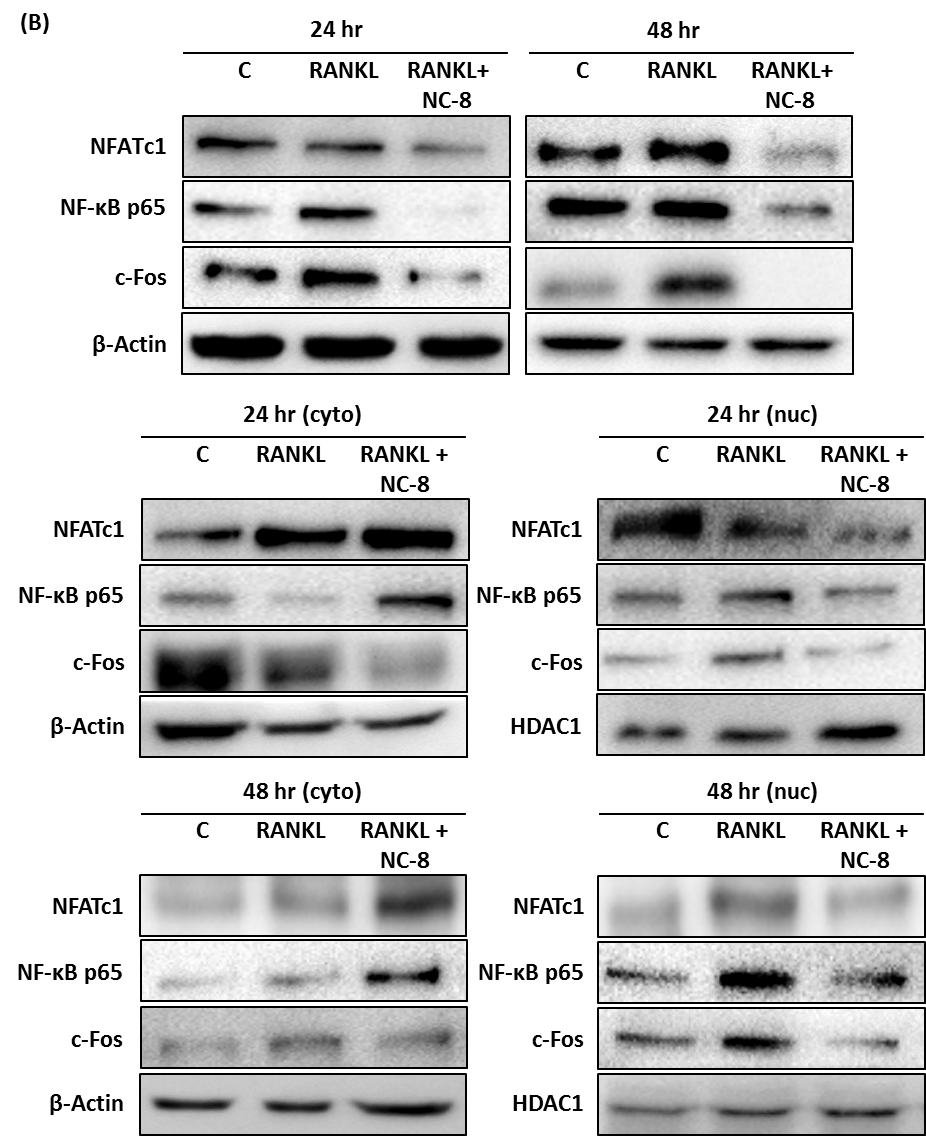


**Supplemental Figure 5. NC-8 inhibited osteoclast differentiation signaling pathway on the RANKL-induced**

RAW 264.7 cells were treated with 100 ng/ml RANKL or with 10 μg/ml NC-8, and the protein was collected after the reaction. Western blot was used to detect MAPK pathway and downstream NFATc1, c-Fos and NF-κB protein molecules. (A) Western blot analysis of proteins related to the MAPK signaling pathway, including p-ERK, p-JNK, and p-p38. β-actin was used as a loading control. (B) Western blot analysis of proteins related to the NF-κB signaling pathway, including p65, NFATc1 and c-Fos. β-actin was used as a loading control. Relative expression levels of p65, NFATc1 and c-Fos protein in the cytoplasm and in the nuclear fractions. β-actin and HDAC1 were used as loading controls.
